# aCGHViewer: A Generic Visualization Tool For aCGH data

**Published:** 2007-02-08

**Authors:** Ganesh Shankar, Michael R. Rossi, Devin E. McQuaid, Jeffrey M. Conroy, Daniel G. Gaile, John K. Cowell, Norma J. Nowak, Ping Liang

**Affiliations:** 1 Department of Cancer Genetics, Roswell Park Cancer Institute, Buffalo, NY 14263 USA;; 2 Department of Biostatistics, The State University of New York at Buffalo, Buffalo, NY 14214 USA.

**Keywords:** array-CGH, CNA, gene expression, visualization

## Abstract

Array-Comparative Genomic Hybridization (aCGH) is a powerful high throughput technology for detecting chromosomal copy number aberrations (CNAs) in cancer, aiming at identifying related critical genes from the affected genomic regions. However, advancing from a dataset with thousands of tabular lines to a few candidate genes can be an onerous and time-consuming process. To expedite the aCGH data analysis process, we have developed a user-friendly aCGH data viewer (aCGHViewer) as a conduit between the aCGH data tables and a genome browser. The data from a given aCGH analysis are displayed in a genomic view comprised of individual chromosome panels which can be rapidly scanned for interesting features. A chromosome panel containing a feature of interest can be selected to launch a detail window for that single chromosome. Selecting a data point of interest in the detail window launches a query to the UCSC or NCBI genome browser to allow the user to explore the gene content in the chromosomal region. Additionally, aCGHViewer can display aCGH and expression array data concurrently to visually correlate the two. aCGHViewer is a stand alone Java visualization application that should be used in conjunction with separate statistical programs. It operates on all major computer platforms and is freely available at http://falcon.roswellpark.org/aCGHview/.

## Background

Array comparative genomic hybridization (aCGH) is a high throughput and high-resolution technique for detecting chromosomal copy number aberrations (CNAs) in the form of deletions, gains, and amplifications of genomic regions or entire chromosomes. aCGH has been widely used to study the association of CNAs with tumorigenesis and tumor progression and to identify specific genes involved in these processes from related regions (Cowell et al 2004; Cowell and Nowak 2003; Deeb et al 2005; Miliaras et al 2005; Nowak et al 2005; Snijders et al 2001). In addition, its application has recently been extended to comparative evolutionary genomics (Locke et al 2003; Watanabe et al 2004) and epigenetic studies (Ching et al 2005; Weber et al 2005). The targets used in aCGH may be bacterial artificial chromosomes (BACs) or other DNA sequences, such as cDNA and oligonucleotides. The number of aCGH analyses performed each year is rising dramatically and with it the need for an interactive viewing platform. This type of experiment typically uses tens of thousands of targets and generates a correspondingly large amount of data. After statistically analyzing the data, aCGH users need the capability to browse through these results in a graphical format, promptly identify the regions demonstrating genomic loss or gain, and quickly access the gene annotation information. aCGH data analysis can normally be very cumbersome and time consuming, and would be facilitated if the data are presented in a graphical rather than tabular format.

To address these issues, many groups have built applications capable of displaying aCGH data. arrayCGHBase (Menten et al 2005), ChARMView (Myers et al 2005; Pinkel and Albertson 2005), CGHAnalyzer (Margolin et al 2005), CGHPRO (Chen et al 2005), CGH-Explorer (Lingjaerde et al 2005), Caryoscope (Awad et al 2004), SeeGH (Chi et al 2004), and M-CGH (Wang et al 2004) are a few of the more recent applications. These groups utilize a variety of approaches, emphasizing different aspects of the data and all having advantages and disadvantages. However, the one characteristic shared by a majority of these tools is the integration of the analytical and visualization functions. While this may seem to be a desirable feature, it actually limits the utility of each tool because of the wide variety of statistical methods available to determine the equivalent copy number for genome segments. These analytical approaches are variously based on Hidden Markov Model (Fridlyand et al 2004), non-parametric change point (Olshen et al 2004), quantile smoothing (Eilers and de Menezes 2005), Bayesian (Daruwala et al 2004), adaptive weights smoothing (Hupe et al 2004), clustering (Wang et al 2005), and heuristic smoothing methods (Jong et al 2004). Each user might prefer to use a particular analysis tool based on experimental purpose and experience. When the visualization function of the software is coupled to the statistical function, the tool is limited to perform only those statistical tests. This limitation prompted us to construct a generic visualization tool that is decoupled from the analytical function and displays aCGH data in a user-friendly, comprehensible, and interactive format.

## Implementation

aCGHViewer is implemented as a user-friendly, standalone Java application. It incorporates JFreeChart as the graphing library and only requires the full Java Development Toolkit (version 1.5 or above; http://java.sun.com/j2se/1.5.0/download.jsp) to execute the program. JFreeChart is an open source Java library and is available at http://www.jfree.org/jfreechart/index.php. aCGH-Viewer has been tested on Windows XP and 2000, Macintosh OS 10.3.9, and Redhat Linux 9. An internet connection is required for launching queries against public databases such as the ones at UCSC Genome Browser or NCBI. aCGHViewer uses the system default web browser on Linux and Macintosh, and Internet Explorer^©^ on Windows. It has been tested to be compatible with Netscape^©^, FireFox^©^, Konqueror^©^, and Safari^©^.

The application is completely de-coupled from any analytical function and accepts input in a simple tab-delimited text file to achieve maximal flexibility. We implemented the program as a desktop application to avoid the latency and performance issues associated with web-based applications. We also avoided a database-backed design to simplify and eliminate the necessity of installing and maintaining a database and web server. Additionally, directing users to the public genomic browser allows them to access up-to-date information and crosslink to other resources.

### Application Features

The features of this application are designed to simplify the process of advancing from a tabular aCGH result set to a limited number of candidate genes. In this process, users usually filter data tables, identify data points of interest, launch a web-based genome browser, and enter the target ID as the search term to retrieve associated genes. aCGHViewer streamlines this process by allowing the user to load datasets, select interesting data points based on graphical information, and then launch a web query using only the mouse.

### Input Format

The input format has been simplified to increase flexibility and interoperability. Nearly all analysis programs available can export data as tab delimited text. aCGHViewer requires input to be in tab delimited text files containing 4 or 5 columns. The 4 column format is intended for ‘uncategorized’ data that has not been classified or flagged into groups based on certain statistical or analytical procedures. The data should be in the format – target ID, chromosome number, target center position, and value. The X-value is the chromosome and center position of the target, and the Y-value can be data from any stage of the aCGH data analysis – raw, intensity-normalized, or statistically-treated but uncategorized. An optional fifth column is used to contain category or flag information. For example, a statistical algorithm might classify each Y-value as belonging to ‘unaffected’, ‘amplified’ or ‘deleted’ groups. aCGH-Viewer currently supports up to 3 categories. The input data can be from any type of experiment or statistical procedure that generates chromosome position dependent values and is not limited to aCGH or expression data. Once the data are successfully loaded, a genomic view is displayed. Multiple data files can be loaded at one time; each is treated as a unique sample, graphed in a separate genomic view and appears as a distinct tab on the main window.

### Genomic View

**Figure 1:**
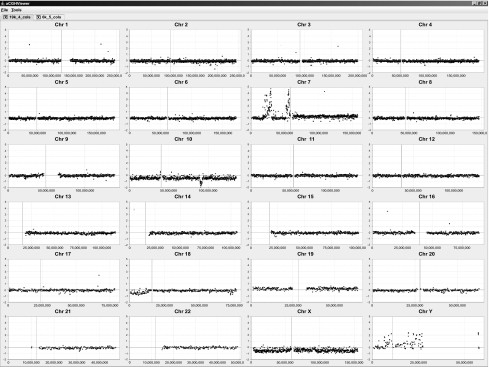
**The aCGHViewer genomic view.** This figure shows the graphical representation of human aCGH data (Rossi et al 2005). Each panel contains the data for one chromosome and each point represents data from one target (BAC). The horizontal axis represents the base pair position along the chromosome while the vertical axis represents the measured log2 signal ratio (−2 – +5) value for each BAC. The position of the centromere is indicated by a vertical black line. Two tabs are visible at the top of the main window in the figure indicating that two data sets have been loaded for visualization.

The genomic view provides the user with a quick method to visually scan all chromosomes and rapidly navigate to the chromosome of interest. The genomic view consists of a set of panels with each containing the graph of one chromosome (Fig. 1). The X-axis of the graph corresponds to the base pair position along a chromosome while the Y-axis corresponds to a measurement value, such as a fluorescence ratio. Selecting a particular chromosome panel launches a resizable detail chromosomal view window that contains only that single chromosome.

### Detailed Chromosomal View

**Figure 2:**
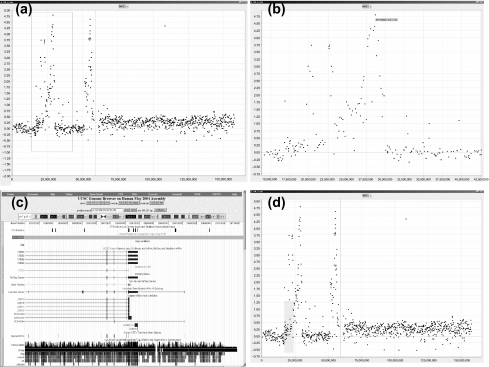
**The detailed chromosome view. (a):** a detailed view launched by selecting chromosome 7 shown in Fig. 1. In this window, the user may zoom in on a portion of the graph by drawing a zooming rectangle surrounding the region of interest. (**b**): a zoom window for a selected region of panel (a) covered by the rectangular box. A tooltip, containing target ID, cytoband, and value, appears when the mouse hovers over a data point of interest. When the data point is selected and clicked, a query is launched against the UCSC or NCBI genome browser. (**c**): the resulting UCSC web page is shown. Note that the target name from the tooltip matches the highlighted target on the webpage. (**d**): a hypothetical breakpoint region within chromosome 7 being selected for exploration. Shift-selecting the suspect region highlights it in yellow and launches a query to the UCSC or NCBI genome browser using the horizontal base pair coordinate range as query parameter. The resulting web page is similar to Fig. 2(c) but showing a larger genomic region.

In the detail window, the user can zoom into an area of interest by click-dragging the mouse in a right-downward motion (Fig. 2a). aCGHViewer automatically fills the detail window with the selected region. The user can zoom out by click-dragging the mouse in a left-upward motion. The zooming scale is dynamic and determined by the area selected by the user. These and other actions are also supported through the right-click menu of the detail window.

The detail chromosomal view supports the central functions of aCGHViewer. When the mouse hovers over a data point, its ID, cytoband, and value are displayed in a tooltip (Fig. 2b) which is helpful if the user needs to confirm that the same target or cytoband is being identified in multiple samples. Selecting a data point launches the genome browser served at UCSC (http://genome.uscs.edu) or NCBI (http://www.ncbi.nlm.nih.gov) with the associated target ID as the query term. The user can then peruse the resulting web page (Fig. 2c) for gene(s) located within the region covered by the target, and use the genome browser website as the gateway to other interlinked resources. Additionally, the user can launch a breakpoint query by selecting a region that visually appears to be at the border of an amplification or deletion (Fig. 2d). In this case, aCGHViewer proffers the chromosome coordinates as the query term to the genome browser, and the resulting web page displays all genes known to be located in the selected region. The user can annotate a data point with its ID, cytoband, and value information by holding down the “Control” key while mouse-clicking the data point (Fig. 4). These annotations help the user to emphasize the location and status of a particular target of interest.

The detail chromosome view window also contains a drop-down menu that can be used to navigate to other chromosomes in the same sample, which obviates the need to search for the genomic view window that may be located behind other windows on the desktop. By displaying multiple detail chromosome view windows from different samples, the user can compare CNA patterns for the same chromosome between samples.

**Figure 3.**
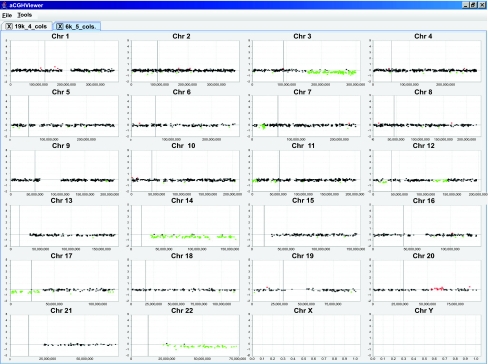
Genome plot of categorized data. Data treated by circular binary segmentation (Olshen et al 2004) are displayed in aCGHViewer. Targets representing amplified regions are colored red, normal regions are in black, and deleted regions are colored green. The X and Y chromosomes in this data set were excluded from analysis because they were utilized as sex-mismatch hybridization controls.

aCGHViewer can also graph categorized or flagged data using the fifth column in the input data file (Fig. 3). Currently, three categories are supported: unchanged, amplified, and deleted are flagged as 1, 2, and 3 respectively. When displayed, targets indicating unchanged copy number are colored black, amplifications are colored red, and deletions are colored green. This type of display helps the user to correlate visual patterns to those generated by the statistical algorithm.

### Simultaneous Plotting of aCGH and Expression Data

**Fig. 4.**
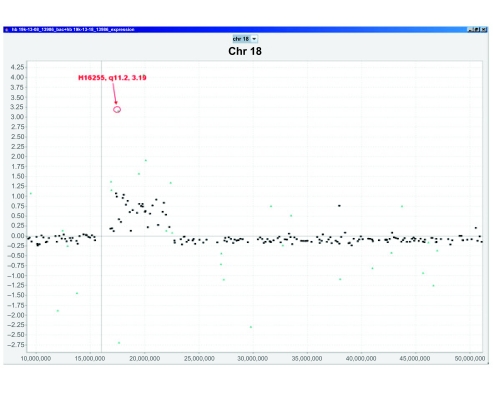
Overlay graph of aCGH and expression data. HNSCC tumor DNA and RNA were analyzed on a RPCI 19K BAC array and 6K cancer specific cDNA array, respectively. A partial region showing amplification by aCGH data (black squares) correlated with elevated expression (magenta triangles) on the q-arm of chromosome 18 was shown in the detailed chromosome view. One particular cDNA spot was annotated with the cDNA ID, cytoband, and log2 value using the annotation function of aCGHViewer (red labels).

Recently, many researchers have generated matching aCGH and expression data for the same sample with the goal of correlating the two types of data. To facilitate this process, we implemented a function in aCGHViewer to support the visualization of aCGH and expression data in a single, overlay graph (Fig. 4). The input format for the expression data is exactly the same as for uncategorized aCGH data. The data are assumed to be in two different files and two file dialogs are displayed. To reduce clutter, the expression data are not displayed in the genomic view but only in the detail chromosome view. The user can zoom, launch probe ID and breakpoint queries in the same manner as for a single plot.

### Program Settings and Options

In its simplest mode of use, aCGHViewer relies on the user’s visual judgement and experience for the identification of CNAs. To further aid the user, the application allows the data to be displayed using 3 different Y-axis scales – experiment relative, absolute value, and chromosome relative. The advantage of using the experiment relative setting is that the maximum and minimum Y-axis values are normalized to the sample level within the experiment, and no data points can be missed. Differences in copy number are also more discernable in this view than in the other two. The disadvantage is that comparison between samples is not easy as each genomic view may have a different scale, thus the user must mentally correct for the different range of values. The absolute setting uses an arbitrary scale that can be defined by the user. This setting lets the user easily compare results between samples but some data points might be missed if they occur outside the user-defined scale. One approach to avoid missing points in the absolute scale is to use the line plot rather than scatter plot setting. This results in a line drawn past the graph boundary and is indicative of a data point lying outside the current viewing limits. The absolute value scale will also tend to de-emphasize the differences within a sample if the absolute range is chosen to be much larger than the actual scale of the data. The default range for the absolute setting is −2 to +5 in log2 signal ratio. The chromosome relative setting plots each chromosome relative to its own scale. This is useful for visualizing the tightness of the data but the borders of an amplified or deleted region may be harder to discern. The user is able to switch between these views at will by using the options dialog.

A number of functions are available from the right-click (context) menu in the detailed chromosome view window. The user can zoom in and out, print the detailed view or save the graph in portable network graphics (png) format. The user may also specify various aspects of the chart, such as title, legend, font, and color. aCGHViewer is able to display any data from any genome that is dependent on chromosome position, but currently can only display the related cytoband information for the mouse and human data. Our intention is to accommodate the cytoband data for more genomes in the future.

## Discussion and Conclusions

aCGHViewer is designed to be a user-friendly visualization tool which is in contrast with existing cumbersome tools that integrate analysis and visualization functions. ChARMView (Myers et al 2005) is the most recently released application and uses the ChARM algorithm to identify breakpoints. Two other tools that were recently released are CGHAnalyzer (Margolin et al 2005) and CGH Explorer (Lingjaerde et al 2005). CGHAnalyzer uses a copy number assignment algorithm while CGH Explorer utilizes bootstrapping and Analysis of Copy Errors (ACE) to classify chromosomal copy number. CGHAnalyzer, based on TIGR’s TM4 (Saeed et al 2003), can also use hierarchical, K-means clustering, and other statistical tests to detect differentially affected regions between groups of samples. All three of these applications are integrated packages that use statistical algorithms as an integral part of their analysis work-flow. These applications are compared by the authors of ChARMView (Myers et al, 2005), who themselves emphasize that manual, visual examination of data is a complementary method that has the advantage of not making any assumptions as any statistical procedure must. arrayCGHBase (Menten et al 2005) is a very complete web-based program that allows for the management and analysis of aCGH experiments and results. The experimental metadata are stored in a MIAME compliant database which is useful for organizing and comparing large projects. However, the program requires the maintenance of a MySQL database, web server and attendant management software.

SeeGH (Chi et al 2004) is one application that most closely matches our focus on visualization. However, its requirement for a local MySQL database, restriction to Windows and inability to display multiple experiments are factors that limit its utility. CGHPRO (Chen et al 2005) is another application with similar dependencies on MySQL and R. Caryoscope (Awad et al 2004) is also a relatively simple visualization tool, but it does not directly link to the external web nor support overlay graphs of aCGH and expression data.

We have built an application that is user friendly, platform independent and compact and supports the workflow of analyzing aCGH data. aCGH-Viewer is purely a visualization tool and should be used in conjunction with separate analytical modules. We have emphasized the use of this tool to analyze aCGH data but it can be extended to analyze expression data or any other types of data which can be converted to associate with a genome position. aCGHViewer is available for the Windows, OS X, and Linux platforms from http://falcon.roswellpark.org/aCGHview/ under the LGPL license. We welcome bug reports and suggestions for improvement or new features from users.

## Figures and Tables

**Table 1.** An example of aCGHViewer input data format for categorized data

BAC_ID^1^	CHR^2^	Position^3^	Value^4^	Category^5^
RP11-430E19	chr1	101448.5	0.409	1
RP11-671C15	chr1	1368581	0.021	1
RP11-201E15	chr1	2738737.5	0.147	1
RP11-41H8	chr1	2738737.5	−0.12	1
RP11-82D16	chr1	3257259	−0.046	1
RP11-62M23	chr1	5477976	−0.069	1
RP11-237N15	chr1	6312316.5	−0.169	1
RP11-51D17	chr1	6285276	−0.112	1
RP11-111O5	chr1	6776316.5	−0.027	1
RP11-58A11	chr1	9723489.5	−0.167	1
RP11-60J11	chr1	10657945.5	0.015	1
RP11-81J7	chr1	13354084	−0.054	1
RP11-874A11	chr1	15620986	0.002	1
RP11-199O1	chr1	15762651.5	0.036	1

^1^ Column for target unique identifiers (human BACs from the RP11 library in this instance);

^2^ Chromosome on which the target is located and formatted as ‘chr#’;

^3^ Center base pair position of the target;

4 Measured value for a target, such as log2 ratio in this example;

5 Optional category flag indicating different classification of data points. Lines without a position or value will be discarded by aCGHViewer. Lines with no explicit category are grouped into the ‘unchanged’ or ‘normal’ category.
